# Characterization of viscerofugal neurons in human colon by retrograde tracing and multi-layer immunohistochemistry

**DOI:** 10.3389/fnins.2023.1313057

**Published:** 2024-01-16

**Authors:** Bao Nan Chen, Adam G. Humenick, Timothy James Hibberd, Wai Ping Yew, David A. Wattchow, Phil G. Dinning, Marcello Costa, Nick J. Spencer, Simon J. H. Brookes

**Affiliations:** ^1^Human Physiology, Medical Bioscience, College of Medicine and Public Health, Flinders University, Adelaide, SA, Australia; ^2^Department of Surgery, Flinders Medical Centre, Bedford Park, SA, Australia

**Keywords:** enteric nervous system, viscerofugal, intestinofugal, multiplexed immunohistochemistry, colon

## Abstract

**Background and Aims:**

Viscerofugal neurons (VFNs) have cell bodies in the myenteric plexus and axons that project to sympathetic prevertebral ganglia. In animals they activate sympathetic motility reflexes and may modulate glucose metabolism and feeding. We used rapid retrograde tracing from colonic nerves to identify VFNs in human colon for the first time, using *ex vivo* preparations with multi-layer immunohistochemistry.

**Methods:**

Colonic nerves were identified in isolated preparations of human colon and set up for axonal tracing with biotinamide. After fixation, labeled VFN cell bodies were subjected to multiplexed immunohistochemistry for 12 established nerve cell body markers.

**Results:**

Biotinamide tracing filled 903 viscerofugal nerve cell bodies (*n* = 23), most of which (85%) had axons projecting orally before entering colonic nerves. Morphologically, 97% of VFNs were uni-axonal. Of 215 VFNs studied in detail, 89% expressed ChAT, 13% NOS, 13% calbindin, 9% enkephalin, 7% substance P and 0 of 123 VFNs expressed CART. Few VFNs contained calretinin, VIP, 5HT, CGRP, or NPY. VFNs were often surrounded by dense baskets of axonal varicosities, probably reflecting patterns of connectivity; VAChT+ (cholinergic), SP+ and ENK+ varicosities were most abundant around them. Human VFNs were diverse; showing 27 combinations of immunohistochemical markers, 4 morphological types and a wide range of cell body sizes. However, 69% showed chemical coding, axonal projections, soma-dendritic morphology and connectivity similar to enteric excitatory motor neurons.

**Conclusion:**

Viscerofugal neurons are present in human colon and show very diverse combinations of features. High proportions express ChAT, consistent with cholinergic synaptic outputs onto postganglionic sympathetic neurons in prevertebral ganglia.

## 1 Introduction

Functional experiments in the 1940s demonstrated that enteric circuits could activate sympathetic neurons in prevertebral ganglia, thus mediating “intestino-intestinal” motility reflexes, without central nervous system input (Kuntz and Saccomanno, 1944). This was the first evidence for enteric “viscerofugal” neurons (VFNs) which are the only enteric neurons that project out of the gut wall. In animal models, synaptic transmission from VFNs to sympathetic post-ganglionic efferent cell bodies is principally mediated by nicotinic-cholinergic receptors (Crowcroft et al., 1971), but may also involve neuropeptides such as VIP (Love and Szurszewski, 1987). The sympathetic targets of enteric VFNs include gut-projecting motor and secretomotor sympatho-inhibitory neurons which release noradrenaline in the gut wall, playing roles in regulating motility and secretion (Macrae et al., 1986). These peripheral reflexes are distinct from the spinal and supraspinal mechanisms that activate sympathetic inhibitory pathways in response to noxious stimuli (Bauer and Boeckxstaens, 2004; Lomax et al., 2010). VFNs comprise fewer than 1% of myenteric neurons in guinea pig small intestine (Costa et al., 1996), although their density increases distally (Messenger and Furness, 1992, 1993; Luckensmeyer and Keast, 1995). VFNs are directly activated mechanically by gut distension (Hibberd et al., 2012a,b) but are also activated synaptically, by other enteric neurons (Sharkey et al., 1998). Recent studies in mice suggest that VFNs may mediate cross-organ signaling between the liver, pancreas and intestines via their projections to sympathetic neurons which, in turn, regulate blood glucose (Muller et al., 2020), and appetite (Zhang et al., 2022).

Viscerofugal neurons have been identified in animal models (rats, mice, guinea-pigs, pigs) but not human gastrointestinal tract. Here, rapid biotinamide neuronal tracing of axons in human colonic nerves *ex vivo* was established, revealing VFNs in the myenteric plexus. A sample of viscerofugal nerve cell bodies (215 neurons, *n* = 6) was characterized in detail, using a multiplexed immunohistochemical protocol, with 12 immunohistochemical nerve cell body markers that distinguish classes of human enteric neurons (Chen et al., 2023).

## 2 Materials and methods

Twenty-five preparations of live human large intestine were taken from the uninvolved margins of excised colon from 23 patients (7 men, 16 women) undergoing surgery for cancer (*n* = 18; aged 31–89 years, median 64 years), polyps (*n* = 2; aged 36 and 38 years), diverticulitis (*n* = 2; aged 53 and 65 years) or Crohn’s disease (*n* = 1; aged 31 years). Supplementary Table 1 summarizes preparations used in this study. All patients signed a written consent form agreeing to donation of tissue for the purpose of research (Southern Adelaide Clinical Human Research Ethics approval number 207.17). Specimens were taken from macroscopically normal tissue at least 5 cm from the edge of the tumor.

### 2.1 Biotinamide tracing

**FIGURE 1:**
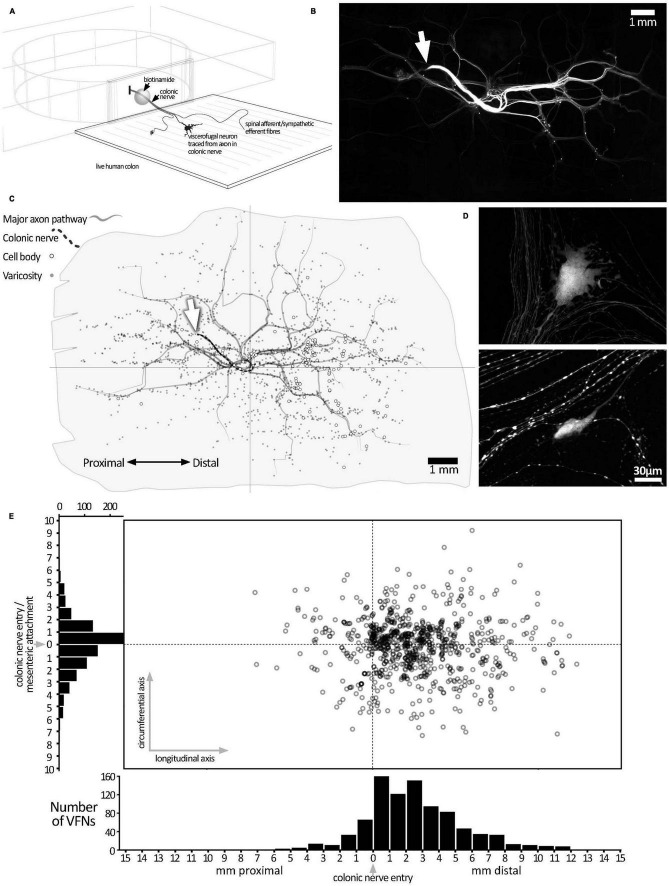
Biotinamide labeling from extrinsic colonic nerve trunks in human colon. **(A)** Schematic diagram of the biotinamide labeling method used to label VFNs in live human colon from colonic nerve trunks. Biotinamide solution was applied as a droplet to a dissected nerve trunk drawn into a perspex chamber filled with paraffin oil and sealed with a coverslip. **(B)** Low power photomontage showing biotinamide filling: the stump of the filled colonic nerve trunk is shown (white arrow). Numerous varicose axons that took up biotinamide can be seen ramifying throughout the myenteric plexus. In addition, numerous enteric nerve cell bodies (VFNs) were labeled (small fluorescent dots). **(C)** A map of the preparation shown in panel **(B)** above, with the locations of 121 VFN cell bodies marked (open circles). Major axon pathways in the myenteric plexus are shown as gray lines and varicose axons branching in ganglia (small gray dots) define the extent of the filled area. Note that VFNs are more numerous aboral to the colonic nerve entry point (at the origin of the axes). **(D)** Higher power confocal images showing typical examples of biotinamide-labeled viscerofugal nerve cell bodies and biotinamide-labeled nerve fibers nearby. **(E)** The locations of 903 viscerofugal nerve cell bodies (open circles) from 25 preparations are shown, mapped relative to the colonic nerve entry point (at the origin of the axes). Viscerofugal nerve cell bodies were concentrated on the aboral side, indicating that their axons usually project orally before exiting the gut wall.

Specimens of live human large intestine were transported to the laboratory in modified Krebs solution gassed with 95% O_2/_5% CO_2_ (20–25°C; in mMol/L: NaCl 118; KCl 4.7, NaH_2_PO_4_ 1; NaHCO_3_ 25; MgCl_2_ 1.2; D-Glucose 11; CaCl_2_ 2.5) and placed in a Sylgard-lined Petri dish (Dow Corning, Midland MI) where they were opened into flat sheets and the mucosa and submucosa were removed. Extrinsic/colonic nerve trunks 5–10 mm long were dissected free from surrounding mesentery and pulled into a nearby small chamber (1 ml volume) sealed with a coverslip and silicon grease (schematic diagram, Figure 1A). The small chamber was then filled with paraffin oil. A bubble of biotinamide solution (5% biotinamide (N-[2-aminoethyl] hydrobromide), Molecular Probes, Eugene, OR), dissolved in artificial intracellular solution [150 mmol/L monopotassium L-glutamic acid, 7 mmol/L MgCl_2_, 5 mmol/L glucose, 1 mmol/L ethylene glycolbis(b-aminoethyl ether)-N,N,N’,N’-tetra-acetic acid, 20 mmol/L Hepes buffer, 5 mmol/L disodium adenosine triphosphate, 0.02% saponin, 1% dimethyl sulfoxide, 100 IU/mL penicillin, 100 lg/mL streptomycin, and 20 g/mL gentamycin sulfate] was placed on the nerve trunk in the small chamber and Krebs solution in the main chamber was replaced with sterile culture medium (Dulbecco’s modified Eagle’s DME/Ham’s F12, Sigma-Aldrich (St Louis, MI) 1:1 ratio mix, supplemented with L-glutamine and 15 mM HEPES; with 10% fetal bovine serum, 1.8 mM CaCl_2_, 100 IU/mL penicillin, 100 g/mL streptomycin D, 2.5 g/mL amphotericin B, 20 g/mL gentamycin, Cytosystems, Castle Hill, NSW, Australia) (Tassicker et al., 1999).

The preparations were incubated overnight (12–16 h; 36°C, 5% CO_2_ in humidified air) with gentle agitation. For immunohistochemical analysis, culture media included 83 μM colchicine (Sigma-Aldrich) to enhance neuropeptide immunofluorescence in nerve cell bodies. Additionally, 5-HT immunofluorescence in cell bodies was enhanced by 5-HT loading (Wardell et al., 1994) during the final 90 min of organ culture by first adding the monoamine oxidase B inhibitor, pargyline (Sigma-Aldrich; 5 × 10^–5^M), followed 30 min later by 5-HT mesylate (Sigma-Aldrich) 2 × 10^–6^M and incubating for 1 h before fixation. Preparations were fixed in 4% paraformaldehyde for 2 nights, then washed and dissected in PBS to remove circular muscle before permeabilizing overnight in PBS containing 0.5% Triton X100 at room temperature. Biotinamide-labeled structures were revealed by overnight incubation in either 3-1-O-(2-cyanoethyl)-(N,N diisopropyl) indo-carbocyanine (CY3)-conjugated streptavidin or Alexa Fluor 488-conjugated streptavidin.

### 2.2 Multiplexed immunohistochemistry

Immunolabeling was carried out with 7 layers of immunohistochemical double-labeling, separated by bouts of antibody elution. Importantly, biotinamide-streptavidin labeled structures were very resistant to elution, allowing each viscerofugal nerve cell body to be identified in each of the 7 layers of staining and be assessed for expression of all 14 markers. For each layer of double labeling, preparations were incubated in mixed primary antisera diluted in hypertonic PBS, at room temperature for 3 days, followed by PBS washes, followed by secondary antisera overnight, then further PBS washes. Primary and secondary antisera are summarized in Supplementary Table 2. Preparations were immersed in bicarbonate buffered glycerol (pH 8.6) then mounted and coverslipped. After analysis and photography, preparations were unmounted and bound antisera were eluted using 50 mls of an aqueous solution comprising 2% w/v sodium dodecyl sulfate (SDS), 62.5 mM Tris–HCl (pH 6.8) and 0.8% 2-mercapto-ethanol, at 56°C for 60 min (Gendusa et al., 2014; Chen et al., 2023) with agitation. Preparations were then remounted and photographed with bracketed exposures to confirm full removal of antisera. They were then unmounted and the next iteration of immunohistochemical double labeling was initiated.

### 2.3 Microscopy and preparation mapping

Preparations were viewed on an Olympus IX71 microscope equipped with epifluorescence and appropriate filter sets (Chroma Technology Corporation, Bellows Falls, VT). Images were captured by monochrome CoolSNAP ES digital camera (Roper Scientific, Photometrics, Tucson, AZ) using analySIS 5.0 (Soft Imaging System, SA, Australia). The microscope stage was fitted with Mitutoyo linear scales (1 μm resolution), connected via Mitutoyo 2D-ALC Decoder (Mitutoyo Corporation, Kawasaki-Shi, Japan) and RS232/USB connector to a personal computer. Stage coordinates, together with an identifying keypress were downloaded into Microsoft Excel spreadsheets via Bill Redirect Software.^1^ Coordinate data was plotted in Excel, precisely mapping the outline of the preparation, location of biotinamide-labeled viscerofugal nerve cell bodies, presence of varicose axons, major axonal pathways, and the location of the entry-point of the filled extrinsic colonic nerve trunk. Confocal images were taken on an LSM 880 microscope (Carl Zeiss, Oberkochen, Germany) with a 20x objective lens (NA:0.8). Z-stacks were scanned at 1.25 μm steps through the full thickness of preparations, then processed to obtain maximum intensity projections using ImageJ (v1.52a; National Institutes of Health).

### 2.4 Statistics and analysis

Statistical analysis was performed by ANOVA (one-way or two-way), or Student’s two-tailed *t*-test for paired or unpaired data using Prism 8 (GraphPad Software, Inc., La Jolla, CA, USA). Statistical differences were considered significant if *P* < 0.05. All data are presented as mean ± SD unless otherwise stated. Lower case “n” indicates the number of patients.

## 3 Results

### 3.1 Spatial distribution of VFNs

Colonic nerve trunks to human large intestine were successfully filled with biotinamide in 25 preparations (*n* = 23; 8 ascending colon, 10 descending colon, 3 sigmoid colon, 4 rectum, 18 female, average age 60.9 years, range: 31–89 years, 20 tumors, 2 diverticulosis, 2 polyps, 1 Crohn’s Disease–see Supplementary Table 1). Biotinamide tracing (Figure 1A) revealed numerous varicose axons that ramified for many millimeters throughout the myenteric plexus; these are likely to belong mostly to sympathetic, parasympathetic or extrinsic sensory neurons. Biotinamide labeled nerve cell bodies in myenteric ganglia were identified as VFNs. Figure 1B shows a typical pattern of neuronal labeling from a single colonic nerve trunk to the descending colon at low power. Biotinamide-labeled VFN cell bodies and unidentified axons are shown in Figure 1D. Between 4 and 121 viscerofugal nerve cell bodies were labeled per preparation, with a total of 903 cells in 25 preparations. The spatial distribution of viscerofugal nerve cell bodies (903 somata, 25 preparations, *n* = 23) was mapped relative to where the colonic nerve trunk from which their axons were traced entered the colon (single preparation in Figure 1C; collated data in Figure 1E). Viscerofugal nerve cell bodies tended to be located distal to the entry point of the colonic nerve, with 85% of VFN axons (767 of 903) projecting orally for 2.7 ± 1.4 mm in the myenteric plexus before exiting via colonic nerves. The remaining 15% projected aborally for 1.3 ± 1.0 mm (136 cells, of 903). Circumferentially, 92% of VFN cell bodies were located within 4 mm of the point of entry of the colonic nerve. There was no difference in the polarized distributions of filled VFNs between ascending colon, descending colon, sigmoid colon and rectum.

### 3.2 Cell body morphology

**FIGURE 2:**
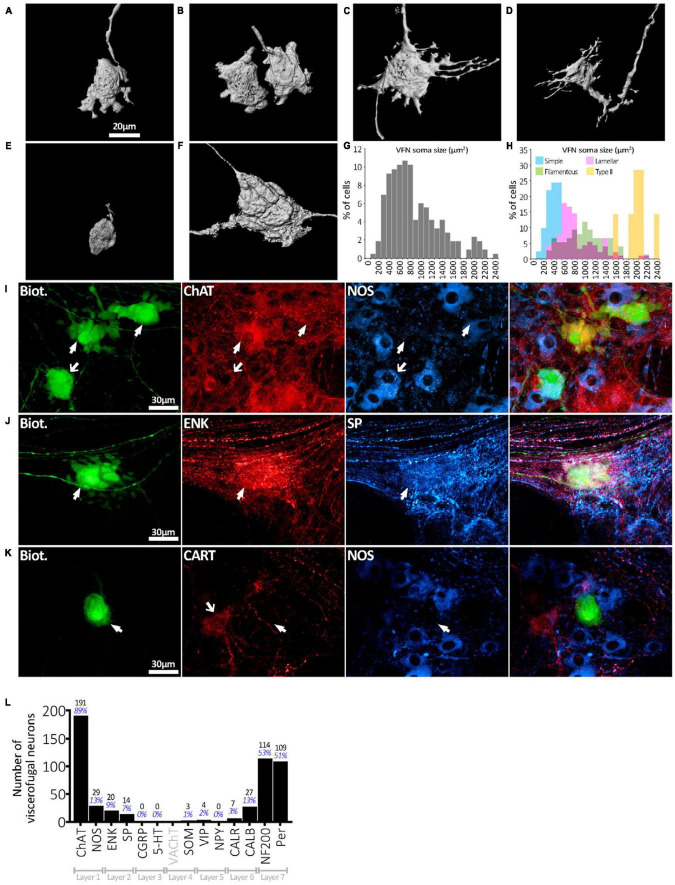
Viscerofugal nerve cell body morphology and neurochemical content. **(A,B)** Three dimensional reconstruction of VFNs; the most common soma-dendritic morphology was uniaxonal with short, broad, flat (“lamellar”) dendrites. **(C,D)** Filamentous VFNs were the second most common morphology; also uniaxonal they have longer spiny dendrites sometimes branching, with or without small lamellae. **(E)** VFN with “simple” morphology was uniaxonal with a small nerve cell body and few or no dendrites. **(F)** Dogiel type II VFN, featuring multiple axons with large nerve cell body, typically with few dendritic features. **(G)** The distribution of nerve cell body sizes among 215 VFNs (*n* = 6) shows a wide range. **(H)** Distribution of nerve cell body sizes among VFNs with different morphological types (215 neurons; *n* = 6). **(I)** Abundant neurochemical markers for VFNs included ChAT, NOS, CALB, ENK and SP and neurofilament markers NF200 and peripherin. The row of matched images in panel **(I)** shows 3 VFNs with ChAT immunoreactivity (arrowheads) and NOS. Solid arrows point to ChAT+/NOS- cells, the open arrow points to a ChAT+/NOS+ cell. **(J)** VFN with ENK and SP immunoreactivities. **(K)** VFN in ganglion lacked immunoreactivity for CART and NOS in its cell body (arrowheads), although other myenteric nerve cell bodies expressed these markers (open arrow). **(L)** Counts and percentages of the sample of 215 viscerofugal nerve cell bodies studied in detail which were immunoreactive for each of the markers. No count is given for VAChT, which only labeled varicosities. Note that many VFNs were immunoreactive for several markers so the percentages shown do not add to 100%.

Six preparations, with 215 biotinamide labeled viscerofugal nerve cell bodies (*n* = 6, 3 ascending colon - 3 female; 3 descending colon-2 female one male–see Supplementary Table 1), were analyzed in detail for soma-dendritic morphology and neurochemistry. The other 19 preparations were used for a study of extrinsic axons innervating the colon. Biotinamide filled the soma-dendritic cytoplasm in detail, allowing labeled cells to be classified by shape. Most VFNs had a single axon (208/215 cells; 97%) with either short lamellar dendrites [mostly corresponding to “stubby cells” according to a previous classification scheme (Brehmer, 2007): 41.9%: 90/215 cells] or filamentous dendrites [corresponding to “spiny cells” (Brehmer, 2007): 35.8%: 77/215 cells] (e.g.: see Figures 2A–D). Some cells had few or no dendrites; (“simple” cells: 19.1%: 41/215 cells; see Figure 2E; Furness et al., 1988). The remaining VFNs (3% 7/215 cells), were multiaxonal Dogiel type II neurons (see Figure 2F; Supplementary Figures 1, 4). The size of viscerofugal nerve cell bodies (as vertical projection area) ranged widely, from 112 to 2,351 μm^2^ (Figure 2G), averaging 878 ± 483 μm^2^ (measured from biotinamide fills). Simple cells were smallest (422 ± 161 μm^2^, 41 cells), followed by lamellar cells (846 ± 382 μm^2^: 90 cells), filamentous cells (1,057 ± 453 μm^2^: 77 cells) and the largest were Dogiel type II (2,006 ± 237 μm^2^: 7 cells). Soma size for each morphological class is summarized in Figure 2H. In summary, in terms of both soma-dendritic morphology and soma size, VFNs showed considerable variety (see Table 1) which was also apparent from NF200 immunoreactivity used in previous studies (Supplementary Figure 9).

**TABLE 1:** Morphology and size of human viscerofugal neurons.

Morphological type	Morphology by biotinamide	% by biotinamide	Average size (μ m^2^)	Std Devn size (μ m^2^)
Simple	41	19.1%	492	148
Filamentous/spiny	77	35.8%	1,209	422
Lamellar/stubby	90	41.9%	1,002	423
Dogiel type II	7	3.3%	2,006	237
Totals	215	100.0%	878	483

A total of 215 biotinamide filled viscerofugal neurons were classified by soma-dendritic morphology as revealed by the cytoplasmic label biotinamide as “simple,” “filamentous,” “lamellar,” or “Dogiel type II”. The size of each morphological type (vertical projection) is also shown. VFNs show considerable variability in morphology and size.

### 3.3 Multiplexed nerve cell body immunolabeling

#### 3.3.1 Nerve cell bodies: single markers

**FIGURE 3:**
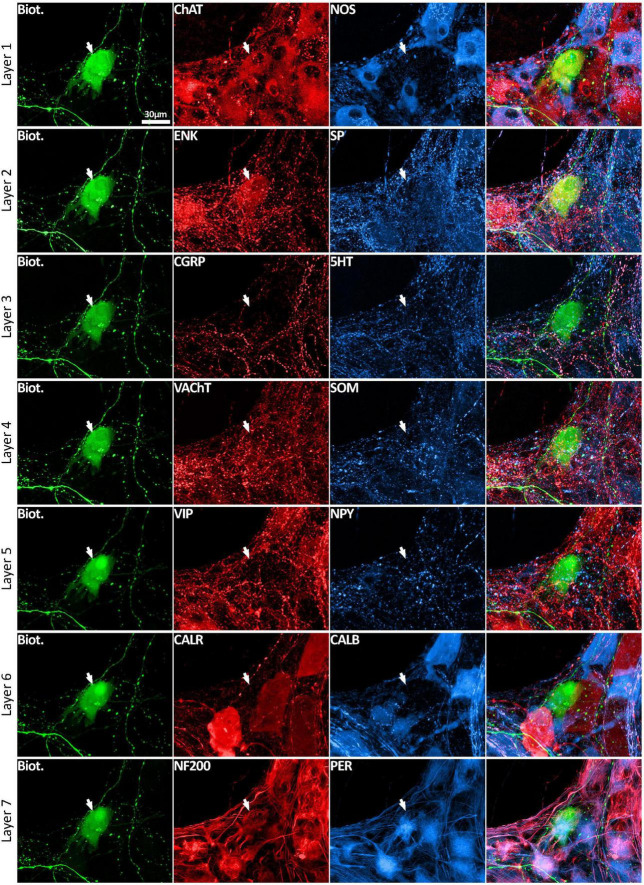
Multiplexed immunolabeling of one VFN. The same viscerofugal nerve cell body, labeled with biotinamide and visualized with streptavidin AF488 is shown here in 7 sequential layers of immunohistochemical double labeling which were separated by an elution step (see methods). This VFN was ChAT+ and ENK+ and NF200+ and Peripherin+ but was non-immunoreactive for all of the other markers. All images are confocal maximum projections. A total of 215 VFNs (*n* = 6) were analyzed in this way.

All 215 VFNs (*n* = 6) were assessed for 12 immunohistochemical discriminating cell body markers (NOS, ChAT, 5-HT, SP, ENK, CGRP, SOM, Calb, Calret, NF200, VIP, NPY) as used in a recent study of human colonic myenteric neurons (Chen et al., 2023). ChAT was the most abundant marker (191/215, 89%; example in Figure 2I), which suggests that human VFNs are principally cholinergic. Smaller proportions of VFNs contained NOS (29/215, 13%; Figure 2I), CALB (27/215, 13%), ENK (20/215, 9%; Figure 2J), SP (14/215, 7%; Figure 2J), CALR (7/215, 3%), VIP (4/215, 2%), SOM (3/215, 1%). No VFN cell bodies were detected with immunoreactivity for 5HT, CGRP or NPY. About half of all VFNs were labeled by antisera for the cytoskeletal proteins NF200 (114/215, 53%) and peripherin (109/215 cells; 51%, see Figure 3. Proportions of VFNs immunoreactive for each of these markers is summarized in Figure 2L. A recent study has reported that VFNs in the mouse are immunoreactive for Cocaine and Amphetamine-Regulated Transcript (CART) (Muller et al., 2020). We tested CART immunoreactivity in 3 preparations of human colon (*n* = 3; 1 ascending colon, 2 descending colon) after filling VFNs with biotinamide applied to colonic nerves. Of 123 VFN cell bodies labeled, none was immunoreactive for CART, although CART-immunoreactive cell bodies and axons were present in most ganglia (Figure 2K and Supplementary Figure 8).

#### 3.3.2 Nerve cell bodies: combinations of markers: “chemical coding”

**FIGURE 4:**
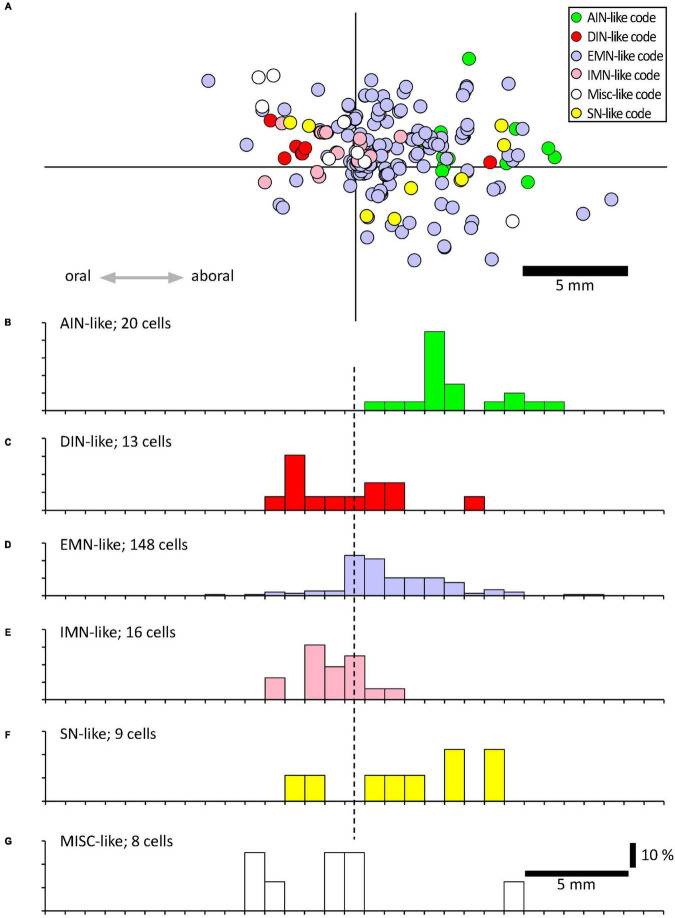
Projections of VFNs with different chemical codes. **(A)** By their combinations of 12 discriminating markers, myenteric neurons have been classified into 20 classes, divided into 6 functional types (Chen et al., 2023); ascending interneurons (AINs); descending interneurons (DINs); excitatory motor neurons (EMNs), inhibitory motor neurons (IMNs), sensory neurons (SNs) and a small miscellaneous type (MISC). **(A)** A total of 215 VFNs showed 27 combinations of markers which allowed 214 of them to be assigned to these 6 types (one did not fit any classification). The location of each cell was mapped relative to the point where their axons projected out of the colon via a colonic nerve (at the origin of the axes). **(B–F)** Each of these types had distinctive axonal projections; EMN-like and AIN-like VFNs (168/214 cells) projected orally to the colonic nerve, similar to EMNs studied previously (Porter et al., 1997; Wattchow et al., 1997; Humenick et al., 2019). DIN-like and IMN-like VFNs were scarcer (29 cells of 214) and showed a slight tendency to project aborally. VFNs with codes of sensory neurons (SN-like, see **F**) or miscellaneous cells (MISC-like, see **G**) were sparse and showed little polarity **(F,G)** .

Using a multi-layer immunohistochemistry protocol (Chen et al., 2023), VFNs showed immunoreactivity for a variety of combinations of markers. Figure 3 shows 7 layers (14 markers), tested in a VFN which was immunoreactive for ChAT, ENK, NF200 and peripherin and lacked the other markers (other examples in Supplementary Figures 1–7). Twelve selective nerve cell body markers, identical to those used in a recent study on human colonic myenteric plexus (Chen et al., 2023) were characterized in 215 VFN cell bodies (*n* = 6). This gives 4,096 possible (2^12^) combinations but in fact, only 27 different combinations were observed among the 215 VFNs. Of these, 26 combinations (accounting for 214 of 215 VFNs) had previously been reported in myenteric neurons of human colon (Chen et al., 2023; see Supplementary Table 3). Just one cell (of 215) had a novel combination of markers not seen in the previous study. Furthermore, comparisons with the same published data (Chen et al., 2023) showed that VFNs had combinations of markers distinguishing all 5 major types of myenteric neurons: excitatory and inhibitory motor neurons (EMNs and IMNs), ascending (orally-projecting) and descending (aborally projecting) interneurons (AINs and DINs) and intrinsic sensory neurons (SNs). However, they were not evenly distributed between these types (ChiSquare = 158.46, df = 5, *p* < 0.001). Over two thirds of VFNs shared the chemical codes belonging to EMNS (Chen et al., 2023) (148 of 215 cells: 68.8%) even though EMNS only comprise 26.1% of all myenteric neurons in human colon (Chen et al., 2023)–see Figures 4A, D. Furthermore, VFNs with these codes mostly had axons projecting orally, as described previously for EMNs (Porter et al., 1997; Wattchow et al., 1997; Humenick et al., 2019). In contrast, VFNs more rarely showed chemical coding defining IMNs (7.4% of VFNs had this coding, where IMNs comprise 41.6% of all myenteric neurons) or DINs (6.0% of VFNs while DINS are 12.3% of all myenteric neurons). Remarkably, VNFs with the coding of IMNs or DINS also shared aborally-directed axonal polarity (see figures 4c, f). The other 3 major types (ascending interneurons, sensory neurons and the miscellaneous group) comprised similar proportions of VFNs compared to all myenteric neurons (data in Table 2 and graphs of polarity in Figure 4B, F, G). There was a significant difference in the proportions of VFNs that expressed combinations of markers that were AIN-like, DIN-like, EMN-like and IMN-like between ascending and descending colon (*X*^2^ = 16.7; df = 3, *P* < 0.001) with a tendency for higher proportions of AINs distally and more EMNs proximally.

**TABLE 2:** Human VFNs share the same chemical codes as multiple types of human myenteric neurons.

Types of cells based on combinations of markers (Chen et al., 2023)	Number of VFNs	% of VFNs (out of 215)	% of all myenteric neurons*	Number of VFNs of each type with ≥ 1 baskets	% of VFNs of each type with ≥ 1 baskets
AIN-like (3 classes combined)	20	9.3%	9.7%	20	100.0%
DIN-like (6 classes combined)	13	6.0%	12.3%	7	53.8%
EMN-like (4 classes combined)	148	68.8%	30.0%	124	83.8%
IMN-like (4 classes combined)	16	7.4%	41.6%	4	25.0%
SN-like (2 classes combined)	9	4.2%	3.5%	7	77.8%
Misc-like (1 class)	8	3.7%	2.9%	7	87.5%
New code (1 cell)	1	0.5%	0%	1	100.0%
Totals	215	99.5%	100.0%	170	

Myenteric neurons in human colon were recently classified into 20 classes of 6 types, based on combinations of 12 immunohistochemical markers (“codes”) that they express (Chen et al., 2023). Of 215 VFNs studied the same way, 9.3% shared codes with ascending interneurons (AINs) which comprise 9.7% of all myenteric neurons. Nearly 69% of VFNs shared codes with excitatory motor neurons, even though these only comprise 30.0% of all myenteric neurons. Fewer VFNs than expected (13.4%) expressed codes of descending interneurons (DINs) and inhibitory motor neurons (IMNs) which together account for 53.9% of all myenteric neurons (Chen et al., 2023). Comparable selectivity was seen when analyzing which VFNs received baskets of varicosities. Every VFN with the chemical coding of an AIN (100%) was surrounded by a basket of varicosities. Likewise most VFNs with coding of EMNs (83.8%; 124 of 148 VFNs) received baskets, whereas few VFNS with coding of IMNs received basket (25%: 4 of 16 cells).

*Data from Chen et al. (2023). Types of neurons in the human colonic myenteric plexus identified by multi-layer immunohistochemical coding. Cell Molec Gastroenterol Hepatol (in press) (Chen et al., 2023).

### 3.4 Baskets of axon varicosities surrounding viscerofugal nerve cell bodies

**FIGURE 5:**
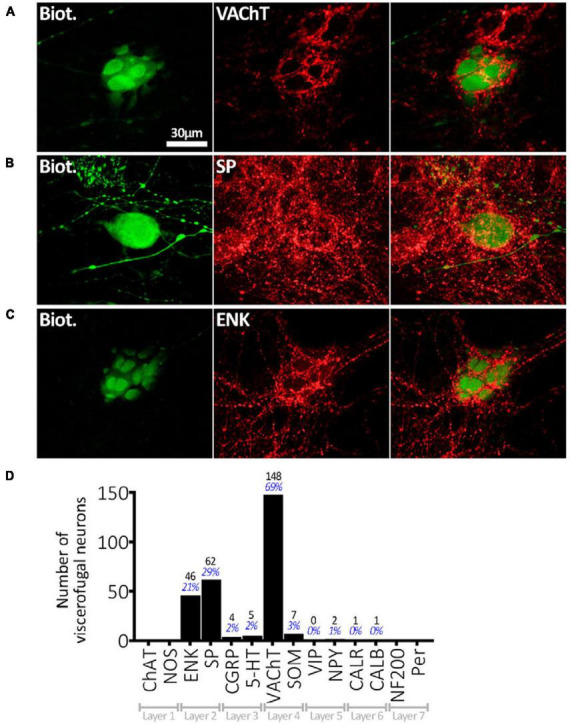
Connectivity of VFNs. **(A)** Biotinamide-filled VFN (green) is surrounded by a cluster (“basket”) of axonal varicosities immunoreactive for VAChT (red) which run in the gap between lamellar dendrites. **(B)** A “simple” biotinamide filled VFN (green) surrounded by a SP-immunoreactive basket. The varicosities form a ring around the cell body. **(C)** Biotinamide filled VFN with clear lamellar dendrites which are closely associated with a basket of enkephalin-immunoreactive axonal varicosities. The presence of lamellar dendrites on the serosal or mucosal face of human myenteric neurons [as shown in panels **(A,C)**] has been described previously (Yew et al., 2023). **(D)** Numbers of 215 VFN cell bodies (*n* = 6) surrounded by baskets with immunoreactivity for each of the 14 markers. VAChT-immunoreactive baskets were most abundant, followed by SP+ and ENK+. The 30 μm scale bar [top left of panel **(A)**] applies to all 9 micrographs. Note that some VFNs received several types of baskets (e.g.: VAChT and SP) and others received none, so the percentages shown do not add to 100%.

Viscerofugal neuron cell bodies were often, but not always, surrounded by distinctive dense rings or clusters of immunohistochemically labeled axonal varicosities, referred to as “baskets,” which looped around the soma and occupied spaces between dendrites (e.g., Figures 5A–C). Baskets could be identified visually by the density of this ring-like-pattern of axonal varicosities, labeled by an immunohistochemical marker, encircling a biotinamide- labeled VFN (Chen et al., 2023). A total of 276 baskets were counted in the sample of 215 neurons (*n* = 6) and most VFNs (170 of 215) received one or more baskets; 76 of 215 cells received 2 or more different types of baskets. The most common baskets were formed by VAChT-immunoreactive varicosities which surrounded 148 of 215 VFNs, (*n* = 7; 68.8%). Varicose axons immunoreactive for SP formed baskets around 62 of 215 cells (28.8%) and ENK-immunoreactive baskets were located around 46 (21.3%). These 3 markers accounted for 92.7% of all baskets; the remaining 7.3% were SOM+, 5HT+, CGRP+, NPY+, CALB+ or CALR+. These data are summarized in Figure 5D. It should be noted that it was not possible to identify reliably baskets formed by extrinsic axons (i.e.: labeled by biotinamide) around intensely labeled VFN cell bodies (also labeled by biotinamide).

Baskets were not randomly distributed between VFNs. For example, VFNs with NOS+ cell bodies were significantly less likely to be associated with any type of basket than NOS- cells [*P* < 0.001, chi square = 60.3, *n* = 215; adjusted standardized residual (ASR) = −4.8]. VAChT+ baskets specifically were sparse amongst NOS+ VFNs (*P* < 0.001, chi squared = 56.3, *n* = 215; ASR = −4.0). = +2.7).

Baskets preferentially targeted some types of VFNs. Viscerofugal neurons with the coding of IMNs (NOS+, ± NF200, ± VIP) (Chen et al., 2023) accounted for 16 of 215 VFNs studied in detail; of these 4 (25.0%) received any basket. In contrast, VFNs with combinations of cell body markers of EMNs (ChAT+ with combinations of ± CALB, ± NF200, ± Calret, ± SOM) accounted for 148 of 215 VFNs studied in detail (i.e.: 68.8%) and of these, 83.8% received one or more baskets. Of VFNs with coding of AINs, 100% received one or more baskets (20 of 20) whereas for VFNs with coding of DINs, 7 of 13 (53.8%) received a basket. Thus baskets preferentially target VFNs with the coding of AINs and EMNs rather than VFNs with markers typical of DINs and IMNs. Interestingly, in a recent study of human myenteric neurons, AINs and EMNs were also preferentially targeted by baskets of varicosities immunoreactive for VAChT, ENK, and SP. This data is summarized in Table 2. It should be noted that some baskets may have arisen from interneurons whose cell bodies were located close to cancerous tissue: we cannot rule out that some baskets may have been affected by this mechanism.

## 4 Discussion

This study identified human colonic viscerofugal neurons (VFNs) by dye-filling them from their axons in colonic nerves, using the tracer biotinamide. VFNs form the neuroanatomical substrate for the afferent arm of intestino-intestinal peripheral sympathetic reflexes in the gastrointestinal tract. Similar to many animal models, most, but not all human colonic VFNs were ChAT-immunoreactive and therefore cholinergic, and had a single axon. Combined neuronal tracing with multiplexed immunolabeling and spatial mapping revealed that VFNs had highly varied combinations of markers, resembling all major types of enteric neurons; no combination of markers (or individual marker) distinguished VFNs from other myenteric neurons. However, over two thirds of VFNs had chemical coding, axonal polarity and putative connectivity indistinguishable from enteric excitatory motor neurons. This raises the possibility that some enteric excitatory motor neurons may be bi-functional and have collateral axons that project to sympathetic ganglia as well as innervating the smooth muscle. Whether VFN axons branch within the gut wall before they exit via the colonic nerves could not be determined in the present study; the dense labeling of sympathetic and other extrinsic axons obscured details of VFN axonal branching. Further studies would be needed to establish whether VFNs innervate targets in the gut wall in addition to prevertebral ganglion neurons.

### 4.1 Comparison with animal models

#### 4.1.1 Location of VFNs

Most human colonic VFN cell bodies in the present study were concentrated within a few millimeters circumferential to the mesenteric attachment; a distribution that is similar to guinea pigs (Kuramoto and Furness, 1989; Luckensmeyer and Keast, 1995, 1996; Linden, 2012), mice (Miller and Szurszewski, 2002) and pigs (Barbiers et al., 1993, 1994). The longitudinal distribution of VFNs, measured relative to where colonic nerve trunks join the bowel, has only been mapped in guinea pig distal colon (Chen et al., 2015) which revealed approximately equal numbers of VFNs with ascending and descending projections. This contrasts with a strong bias toward ascending projections in human VFNs, shown in the present study. However, projections of subtypes of VFNs in the guinea-pig were polarized; ChAT+/NOS+ VFNs in guinea pig distal colon were significantly more likely to have aborally-directed axonal projections than ChAT+/NOS- VFNs (Chen et al., 2015). Descending polarity of NOS-expressing VFNs was also a feature of human colonic VFNs in the present study (Figures 4D, E). It has been reported that some viscerofugal neurons in pig colon have cell bodies located in the submucous plexus (Barbiers et al., 1993). Whether this is true for humans remains to be determined because the submucous plexus had been removed from preparations prior to biotinamide.

#### 4.1.2 Cell body morphology

Nearly all (208/215 cells; 97%) human VFNs were uniaxonal, similar to most studies in laboratory animals in which VFNs retrogradely traced from celiac, superior mesenteric, inferior mesenteric ganglion, or from extrinsic nerve trunks were reported as uniaxonal [guinea pig (Kuramoto and Furness, 1989; Messenger and Furness, 1992, 1993; Sharkey et al., 1998; Tassicker et al., 1999; Lomax et al., 2000; Olsson et al., 2004; Chen et al., 2015), rat (Furness et al., 2000), mouse (Miller and Szurszewski, 2002) and pig (Timmermans et al., 1993)]. In a few studies, small numbers of multiaxonal VFNs have been reported; in the guinea pig (Ermilov et al., 2003; Linden, 2012; Hibberd et al., 2014) and rat (Luckensmeyer and Keast, 1995). Moreover, “lamellar”, “filamentous”, and “simple” morphological subtypes of uniaxonal neurons have all been reported in VFNs in animals (Furness et al., 2000; Miller and Szurszewski, 2002; Chen et al., 2015), similar to the variety seen here in human colonic VFNs.

#### 4.1.3 Neurochemistry of human VFNs

Neurochemically, most human colonic VFNs (89%) were immunoreactive for ChAT and hence likely to be cholinergic, similar to VFNs in guinea pig gut (Mann et al., 1995; Sharkey et al., 1998; Hibberd et al., 2014; Chen et al., 2015) and rat (Furness et al., 2000). This is consistent with acetylcholine being a fast excitatory neurotransmitter released by viscerofugal neurons at synapses onto prevertebral sympathetic post-ganglionic neurons (Crowcroft et al., 1971).

The expression of NOS in a subset of VFNs (13%) is similar to colonic VFNs in guinea pig (Mann et al., 1995; Sharkey et al., 1998; Hibberd et al., 2014; Chen et al., 2015) and rat (Luckensmeyer and Keast, 1996), although proportions were higher in those species (>50% in guinea pig and ∼20–30% in rat). However, only 1% of IMG-projecting myenteric VFNs in pig distal colon contained NOS (Barbiers et al., 1994) suggesting a high degree of variability between species.

The calcium binding protein, calbindin (CALB) is a widespread marker in human colon, labeling over 26% of myenteric neurons (Chen et al., 2023), but was expressed in 13% of human colonic VFNs; a smaller proportion than in the guinea pig (Furness et al., 1990; Messenger and Furness, 1992, 1993; Mann et al., 1995) and rat (Luckensmeyer and Keast, 1996) where most colonic VFNs expressed CALB. However, IMG-projecting VFNs in dog colon did not express CALB (Li and Masuko, 1997). The related calcium-binding protein, calretinin (CALR) was detected in 3% of human VFNs. There is little data for comparison in animal models but in one study of 10 IMG-projecting VFNs tested in guinea pig colon none was CALR-immunoreactive (Sharkey et al., 1998).

Approximately 9% of human colonic VFNs expressed ENK. In the guinea pig, studies using nerve lesions suggested that most VFNs lack ENK (Dalsgaard et al., 1983; Masuko and Chiba, 1988; Webber and Heym, 1988). One study revealed ENK immunohistochemically in canine VFNs but this was not quantified (Li and Masuko, 1997). ENK-immunoreactive varicose nerve fibers are present in human CG and SMG (Jarvi et al., 1988; Schmidt et al., 1990; Del Fiacco et al., 1993; Quartu et al., 1993) consistent with a subset of human VFNs expressing ENK. However, contributions by axons from spinal afferent and sympathetic preganglionic neurons cannot be ruled out (Przewłocki et al., 1983).

Immunoreactivity for substance P or related tachykinins was detected in 7% of human VFNs in the present study whereas in the guinea pig IMG few VFNs express tachykinins (Dalsgaard et al., 1983; Masuko and Chiba, 1988; Webber and Heym, 1988) However, SP was reported in some colonic VFNs in the dog (Li and Masuko, 1997). High densities of SP+ nerve fibers have been reported in all human sympathetic prevertebral ganglia (Hua et al., 1987; Schmidt et al., 1990, 1992; Del Fiacco et al., 1992, 1993; Quartu et al., 1993) in many cases colocalizing with CGRP, suggesting that they arise from spinal afferent fibers (Franco-Cereceda et al., 1987). CGRP and NPY were not detected in human colonic VFNs in the present study.

Four of 215 (2%) human colonic VFNs contained VIP, whereas this peptide is more abundant in several small animal models; guinea small intestine (Kuramoto and Furness, 1989; Mann et al., 1995) large intestine (Furness et al., 1990; Messenger and Furness, 1992, 1993) in rat large intestine (Luckensmeyer and Keast, 1996), as well as in some dog colonic VFNs (Li and Masuko, 1997). However, in the pig, neither small intestinal (Timmermans et al., 1993) nor colonic (Barbiers et al., 1993) VFNs were VIP-immunoreactive. In human CG and SMG, VIP+ fibers are abundant (Schmidt et al., 1990; Del Fiacco et al., 1993; Quartu et al., 1993) and some principle ganglion nerve cell bodies (Del Fiacco et al., 1993; Quartu et al., 1993) and small intensely fluorescent (SIF) cells (Del Fiacco et al., 1993) also express VIP. The results of the present study suggest few VIP+ axons in human CG and SMG originate from colonic VFNs. Possible alternative sources of VIP+ include principal ganglion neurons (Del Fiacco et al., 1993; Quartu et al., 1993) inputs from spinal pre-ganglionic neurons and axon collaterals of spinal afferent neurons (Charnay et al., 1985).

Three of 215 (1%) human colonic VFNs contained SOM but equivalent data is lacking in laboratory animals. SOM+ fibers are present in human CG and SMG (Del Fiacco et al., 1993; Quartu et al., 1993). The present study suggests that few of these are likely to originate from colonic VFNs. More likely sources include SOM+ principal ganglion neurons (Del Fiacco et al., 1993; Quartu et al., 1993) and collaterals of spinal afferent neurons (Nagao et al., 1994). No human colonic VFNs contained detectable 5HT. In the pig, 5HT has been reported in small intestine VFNs (Timmermans et al., 1993) and in 30% of colonic VFNs of the outer submucous plexus (Barbiers et al., 1993), but not the myenteric plexus (Barbiers et al., 1993).

The absence of CART from a sample of 123 VFN cell bodies (*n* = 3 patients) is in contrast to the mouse, where in both small and large intestines VFNs are substantially CART-immunoreactive (Muller et al., 2020). All of these variations in expression reinforce the extent of inter-species differences and indicate the importance of direct analysis of human tissue, wherever possible. The functional significance of the high degree of variability in VFN markers is obscure. However, recent studies in C elegans suggest that non-synaptic release of neuropeptides, coupled with selective expression of receptors in target neurons, can mediate volume transmission that can operate over many microns (Randi et al., 2023; Ripoll-Sanchez et al., 2023). We speculate that neuropeptides released from VFN terminals in prevertebral ganglia may functionally modulate specific sympathetic pathways under appropriate conditions.

### 4.2 Combinations of markers

While single neurochemical markers show considerable variability between species and regions, the combinations of markers in VFNs were informative. A recent study characterized human colonic myenteric neurons using a multiplexed battery of 12 discriminating nerve cell body markers (2,596 neurons, *n* = 12), and divided them into 20 classes (Chen et al., 2023). The same 12 markers were applied to all 215 VFNs detailed in the present study (NOS, ChAT, 5-HT, SP, ENK, CGRP, SOM, Calb, Calret, NF200, VIP, NPY). The most striking finding was that VFNs shared “coding” (i.e.: specific combinations of markers) with all 5 major types of myenteric neurons; EMNs, IMNs, AINs, DINs and SNs. Only one VFN (of 215 sampled) had a novel combination of markers not present in the published account. However, some VFN codes were greatly over-represented: excitatory motor neurons (EMNs) constitute 30% of myenteric neurons (Chen et al., 2023) but VFNs with EMN-like codes made up 68.8% of 215 cells sampled. Cells with aborally-directed projections (descending interneurons, inhibitory motor neurons) made up 53.9% of all myenteric neurons but only accounted for 13.4% of the 215 VFNs. Thus VFNs form a diverse group of neurons, without a singular, distinctive chemical coding, but many of which have neurochemical and anatomical features of excitatory motor neurons. Overall, VFNs show a significant tendency to have orally-projecting axons which is another feature shared by excitatory motor neurons (Wattchow et al., 1997).

### 4.3 Baskets of varicose axons around VFNs

Most human VFNs (68.8%) were surrounded by VAChT+ baskets; consistent with VFNs receiving nicotinic cholinergic synaptic inputs from other enteric neurons, as shown electrophysiologically in mouse and guinea pig (Crowcroft et al., 1971; Sharkey et al., 1998; Miller and Szurszewski, 2002; Hibberd et al., 2014). In guinea pig distal colon, 93% of retrogradely-labeled VFN cell bodies were surrounded by VAChT+ baskets and VIP+ baskets (Lomax et al., 2000). Human colonic VFNs were predominantly surrounded by baskets of VAChT varicosities but received very few VIP baskets. However, ENK and SP baskets were also abundant in human colon. These latter markers probably arise from ascending interneurons (Humenick et al., 2021), and possibly intrinsic sensory neurons (Wattchow et al., 1997; Brehmer et al., 2005; Chen et al., 2023). VAChT, ENK, and SP baskets disproportionately target excitatory motor neurons in human colonic myenteric plexus (Chen et al., 2023). Thus the majority of human VFNs share multiple features with human excitatory motor neurons, namely chemical coding, axon polarity and putative sources of synaptic input (as reflected by baskets). As mentioned above, it cannot be ruled out that some VFNs have dual axonal projections to sympathetic ganglia and to enteric smooth muscle and may also function as excitatory motor neurons. It should also be noted that some baskets around VFNs may have arisen from enteric interneurons whose cell bodies were located close to the tumor. We cannot rule out that some baskets may have been affected by this mechanism.

### 4.4 Roles of VFNs

The existence of VFNs in human colon with the features described above make it possible to speculate about their possible roles. While VFNs are modestly mechanosensitive, their distension-driven firing largely depends on synaptic input from other enteric neurons (Crowcroft et al., 1971; Miller and Szurszewski, 1997). Physiologically-relevant distensions also evoke the well-known ascending excitatory reflex (Bayliss and Starling, 1900) in which excitatory motor neurons are activated according to the “law of the intestine.” We speculate that VFNs are synaptically activated in-parallel with enteric excitatory motor neurons during stimulus-induced motor behavior. This would explain why VFNs fire just prior to muscle shortening, during bouts of spontaneous contractility (Hibberd et al., 2012b). In the mouse, spontaneous colonic motor complexes display 2 Hz rhythmic contractions driven by synchronized firing of cholinergic enteric excitatory motor neurons (Spencer et al., 2005, 2018). Colonic VFNs in mice fire bursts of action potentials phase-linked with this 2 Hz rhythmicity (Hibberd et al., 2020) consistent with VFNs and excitatory motor neurons sharing a common drive. VFN firing during colonic motor complexes continues when the muscle is pharmacologically paralyzed, indicating that synaptic drive from enteric neurons (rather than mechanosensitivity) predominates (Hibberd et al., 2020).

Viscerofugal neurons synaptically activate sympathetic post-ganglionic efferents, which project back to the gut (Hibberd et al., 2020) to inhibit motility, mostly via presynaptic inhibition in the myenteric plexus (Hirst and McKirdy, 1974) although direct effects on muscle also occur (Spencer et al., 1999), which may be relevant in post-operative ileus (Wattchow et al., 2021). Thus, the VFN-mediated reflex pathway, activated in concert with motility circuits, may provide negative feedback that limits excitatory drive to smooth muscle during CMCs. This mechanism could explain the retention of fecal pellets in the colon of rodents *in vivo* and the slower propulsion of pellets *in vivo* compared to *ex vivo* (when sympathetic drive is removed) (Smolilo et al., 2023). VFN activation may also modulate synaptic input from sympathetic pre-ganglionic neurons in prevertebral ganglia (Quinson et al., 2000)and may also coordinate activity between different regions of gut (Lomax et al., 2010). The presence of VFNs in human colon, similar to many other species, suggest that they play an important role in gut physiology that has been evolutionarily conserved across many mammals.

## 5 Conclusion

Studies in a variety of laboratory animals have demonstrated the presence of viscerofugal neurons projecting from the gut to sympathetic pre-vertebral ganglia which contribute significantly to the long-range control of gut motility. The present study has characterized viscerofugal neurons in human colon for the first time. Human VFNs are principally cholinergic neurons with a single axon that projects orally along the gut before exiting through colonic nerves. Most are surrounded by baskets of axonal varicosities arising from other enteric neurons, including those in ascending neural pathways. These are likely to mediate cholinergic excitatory synaptic drive to VFNs as part of reflex control of gut function.

## Data availability statement

The datasets presented in this study can be found in online repositories. The names of the repository/repositories and accession number(s) can be found below: Pennsieve with Doi: 10.26275/liud-lt1z.

## Ethics statement

The studies involving humans were approved by the Southern Adelaide Clinical Human Research Ethics Committee (permit number 207.17). The studies were conducted in accordance with the local legislation and institutional requirements. The participants provided their written informed consent to participate in this study.

## Author contributions

BC: Data curation, Investigation, Methodology, Resources, Visualization, Writing—review and editing. AH: Data curation, Investigation, Methodology, Visualization, Writing—review and editing. TH: Data curation, Formal analysis, Methodology, Writing—original draft, Writing—review and editing. WY: Data curation, Investigation, Methodology, Visualization, Writing—review and editing. DW: Conceptualization, Funding acquisition, Methodology, Resources, Writing—review and editing. PD: Conceptualization, Funding acquisition, Resources, Supervision, Writing—review and editing. MC: Conceptualization, Methodology, Writing—review and editing. NS: Conceptualization, Supervision, Validation, Writing—review and editing. SB: Conceptualization, Formal analysis, Funding acquisition, Methodology, Supervision, Writing—original draft, Writing—review and editing.

## Footnotes

1
www.billproduction.com
