# Supplementary figures and images for: Characterization of viscerofugal neurons in human colon by retrograde tracing and multi-layer immunohistochemistry
Source: Front Neurosci. 2024 Jan 16;17:1313057. doi: 10.3389/fnins.2023.1313057 (PMC10825022; doi:10.3389/fnins.2023.1313057)

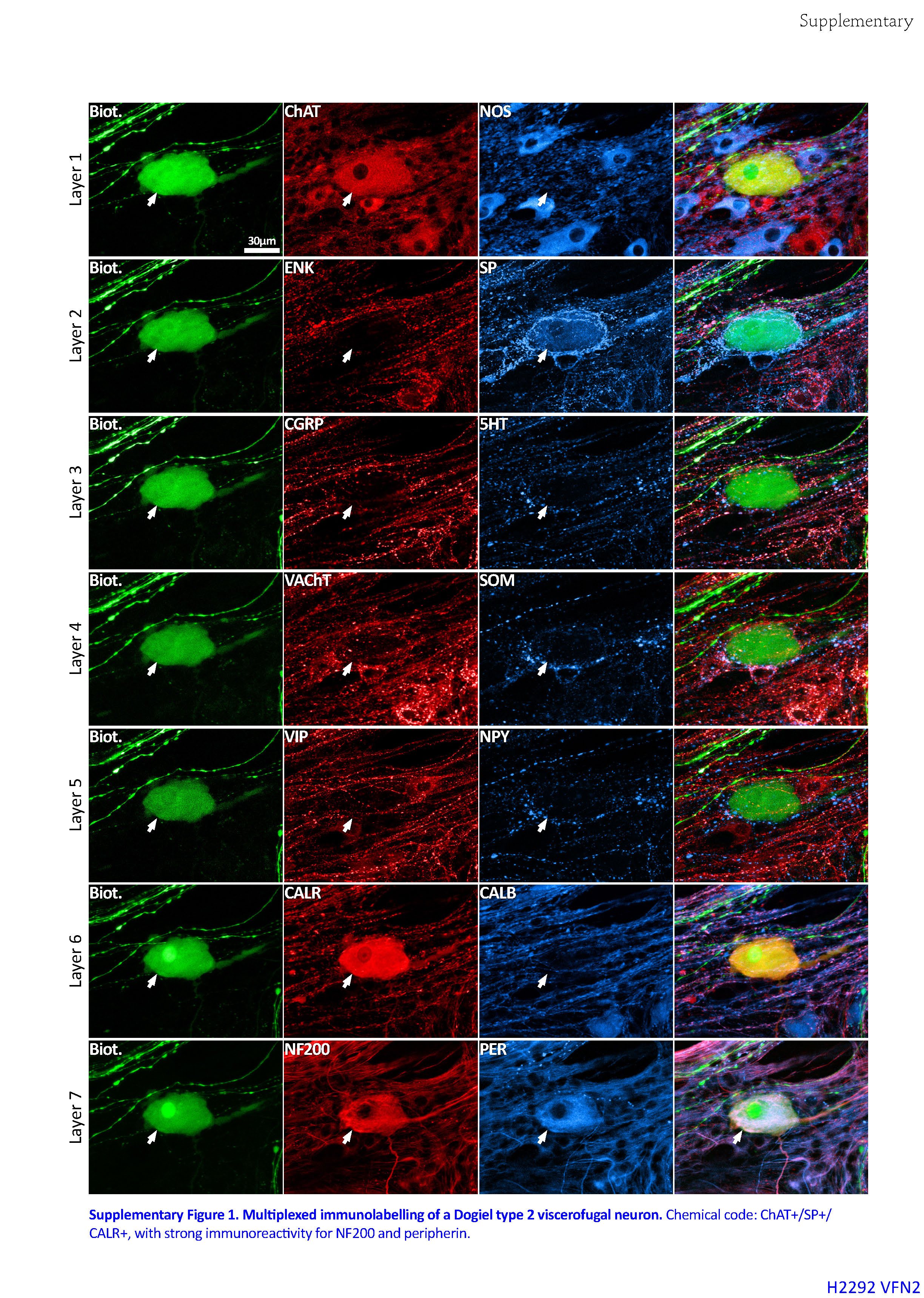

Supplement: Supplementary file 1 [file Image_1.JPEG]

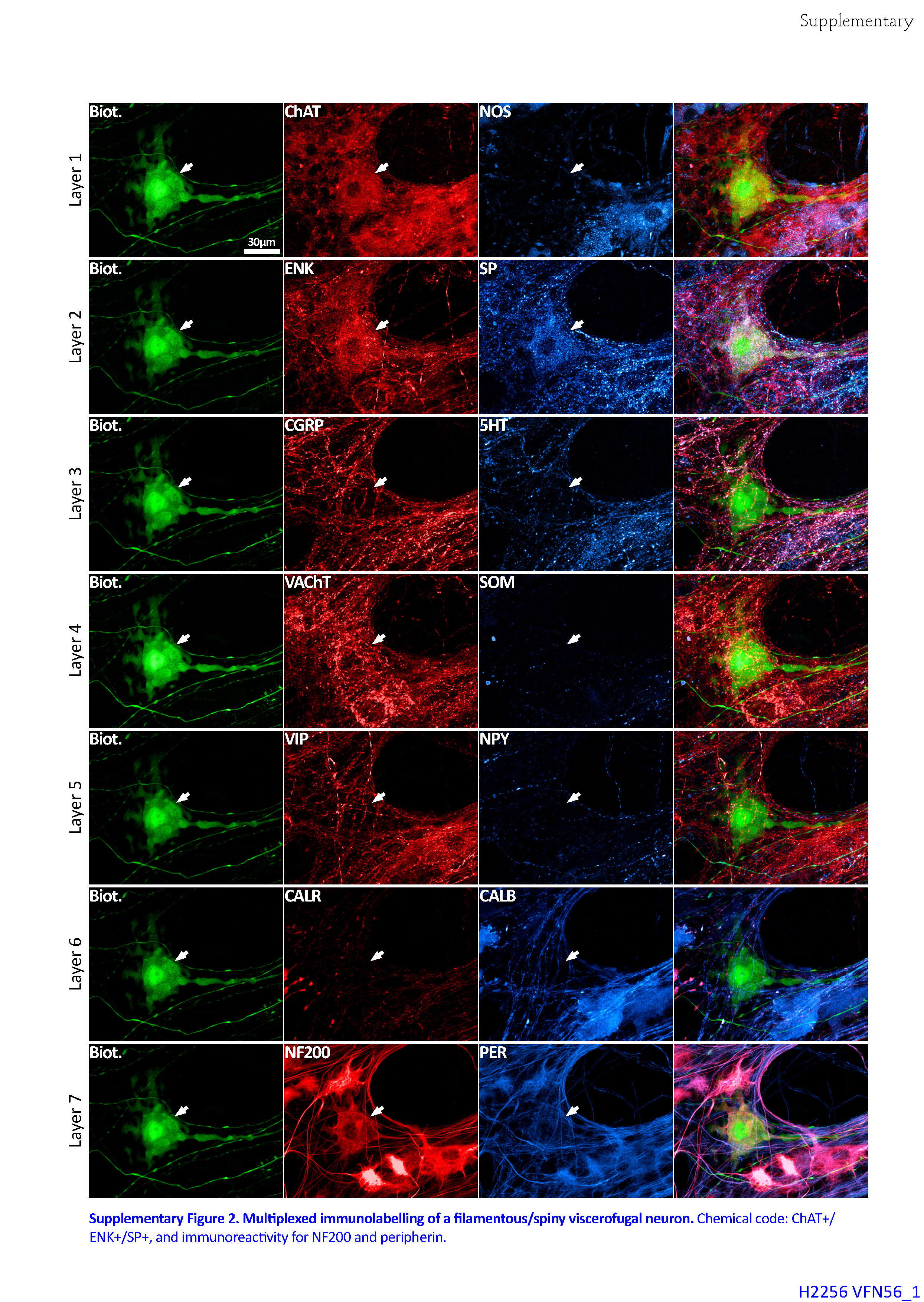

Supplement: Supplementary file 2 [file Image_2.JPEG]

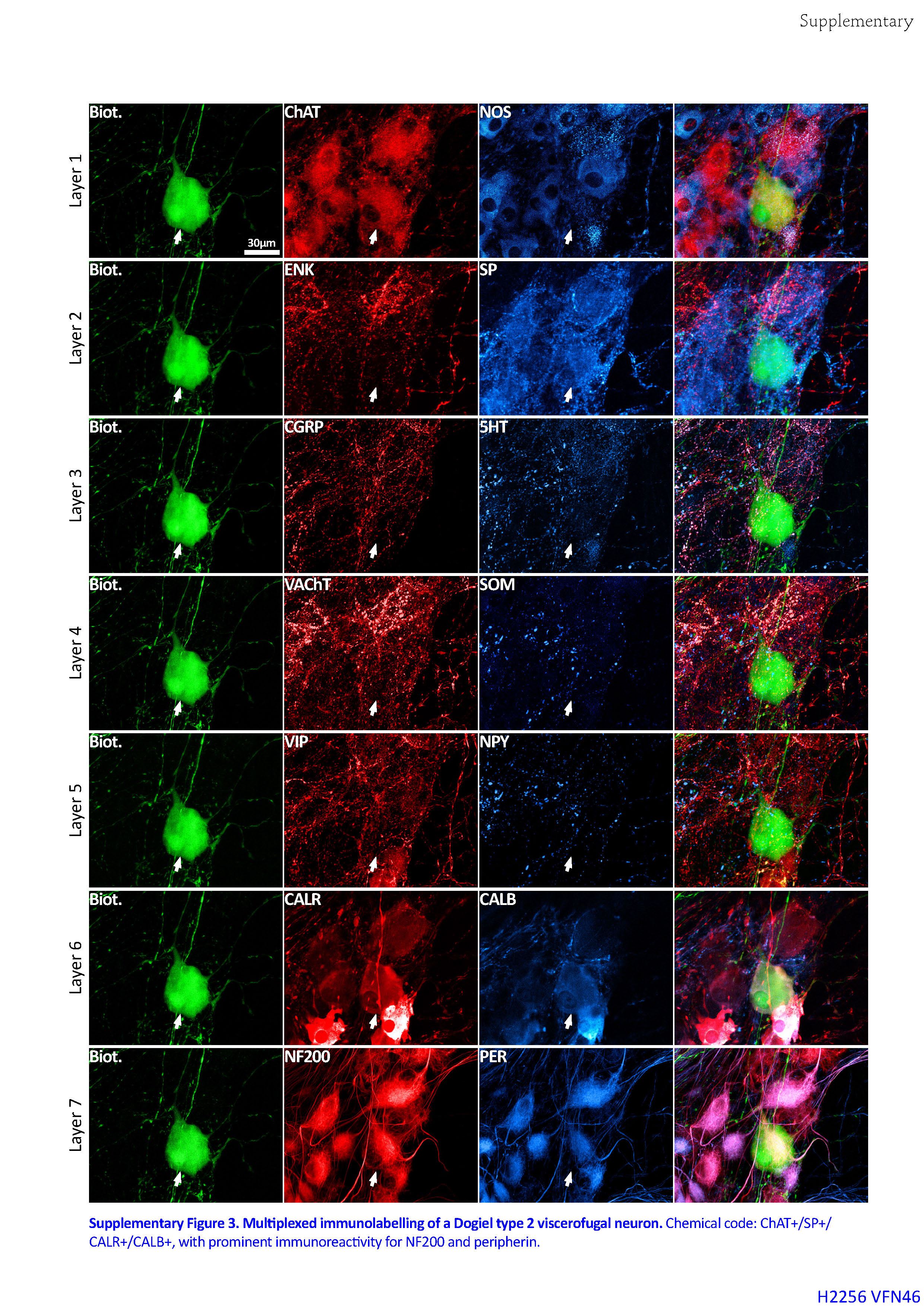

Supplement: Supplementary file 3 [file Image_3.JPEG]

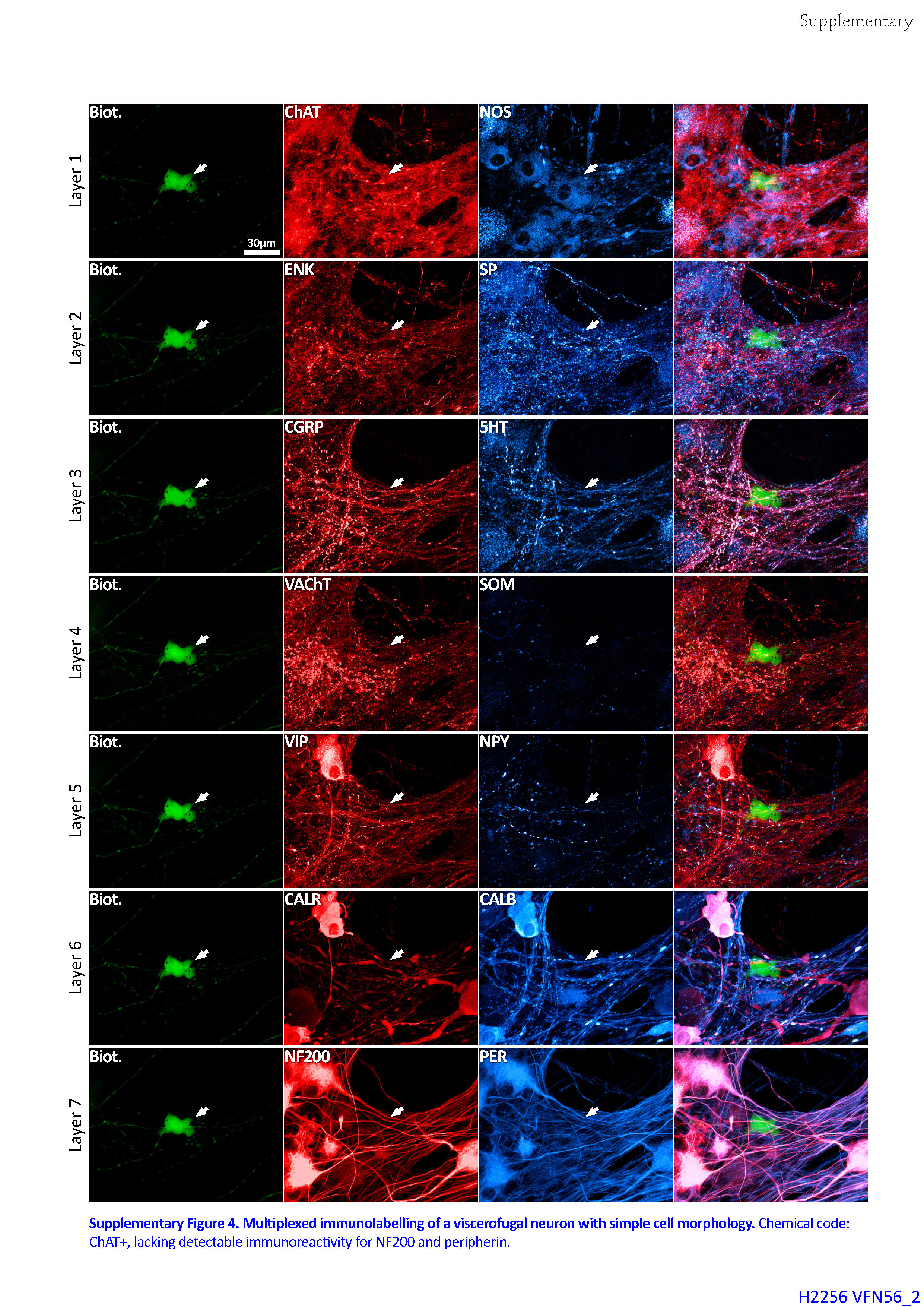

Supplement: Supplementary file 4 [file Image_4.JPEG]

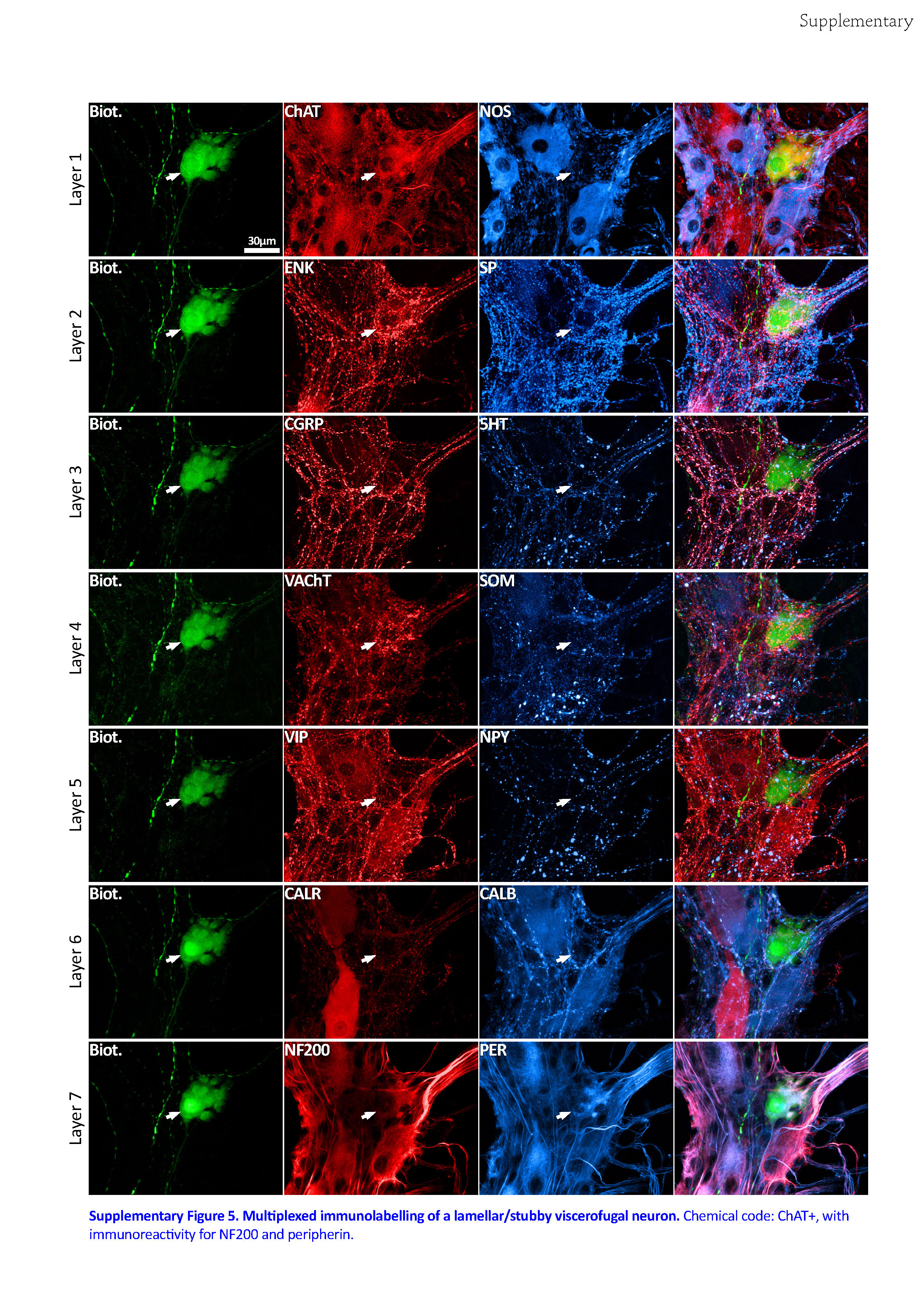

Supplement: Supplementary file 5 [file Image_5.JPEG]

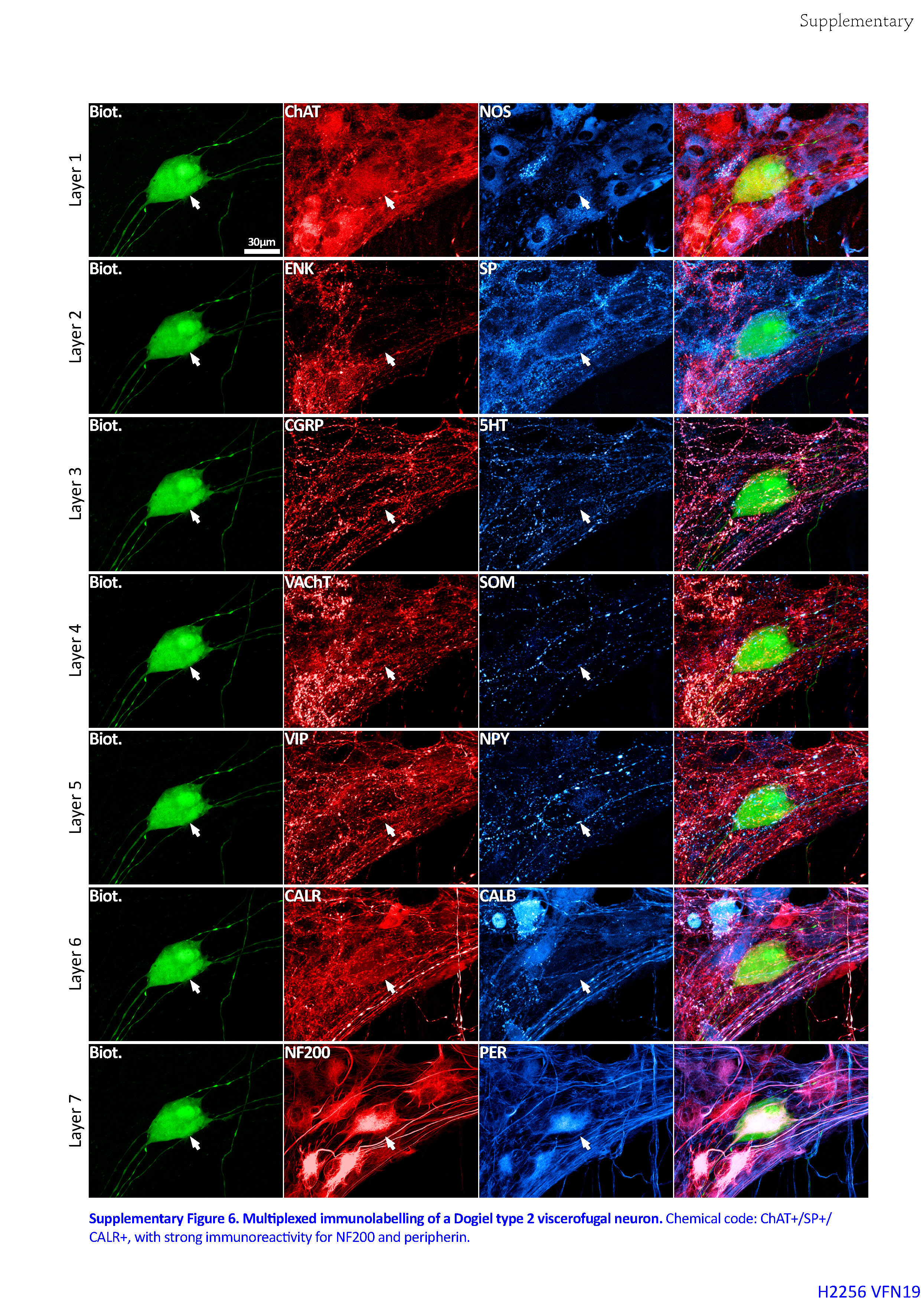

Supplement: Supplementary file 6 [file Image_6.JPEG]

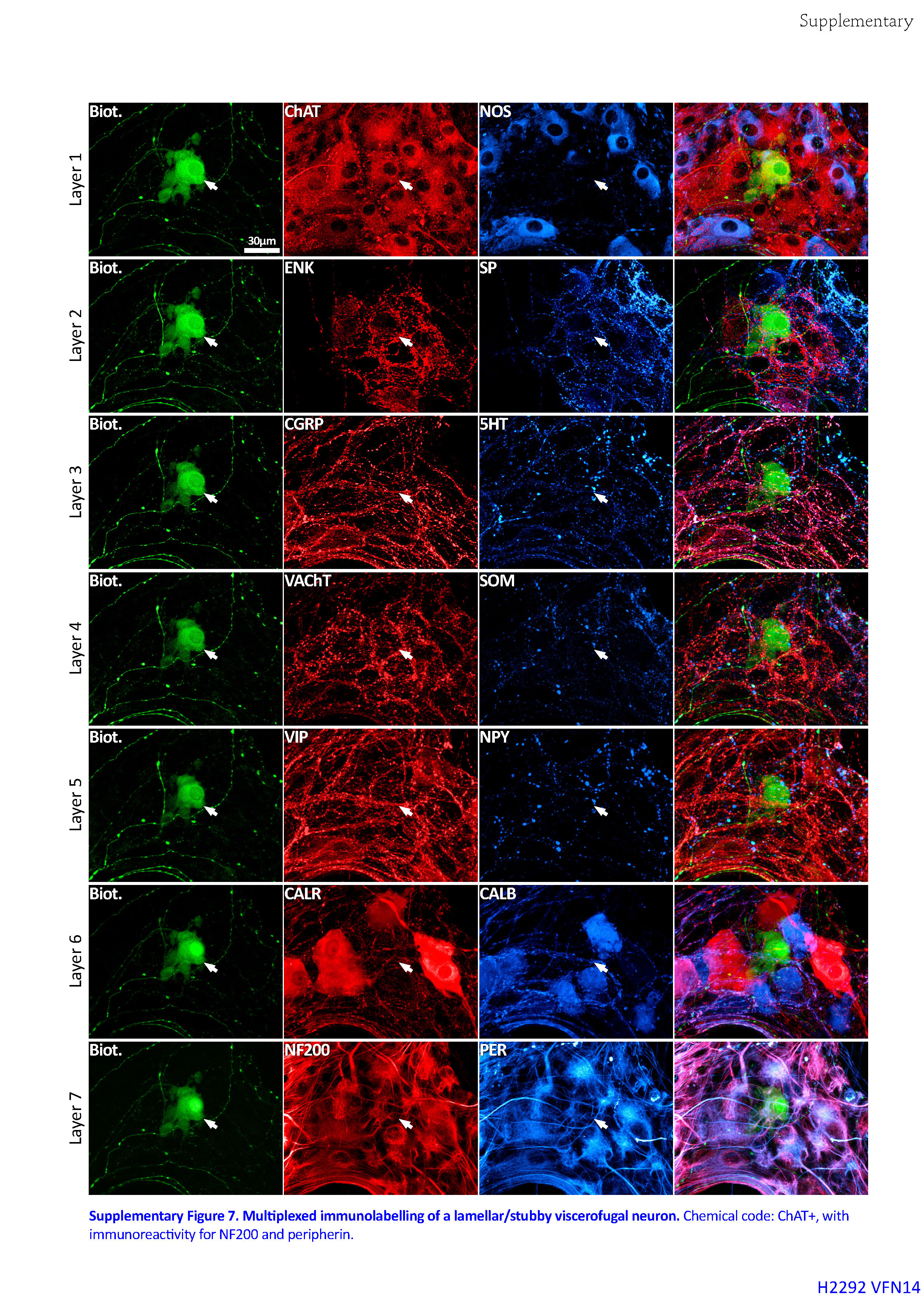

Supplement: Supplementary file 7 [file Image_7.JPEG]

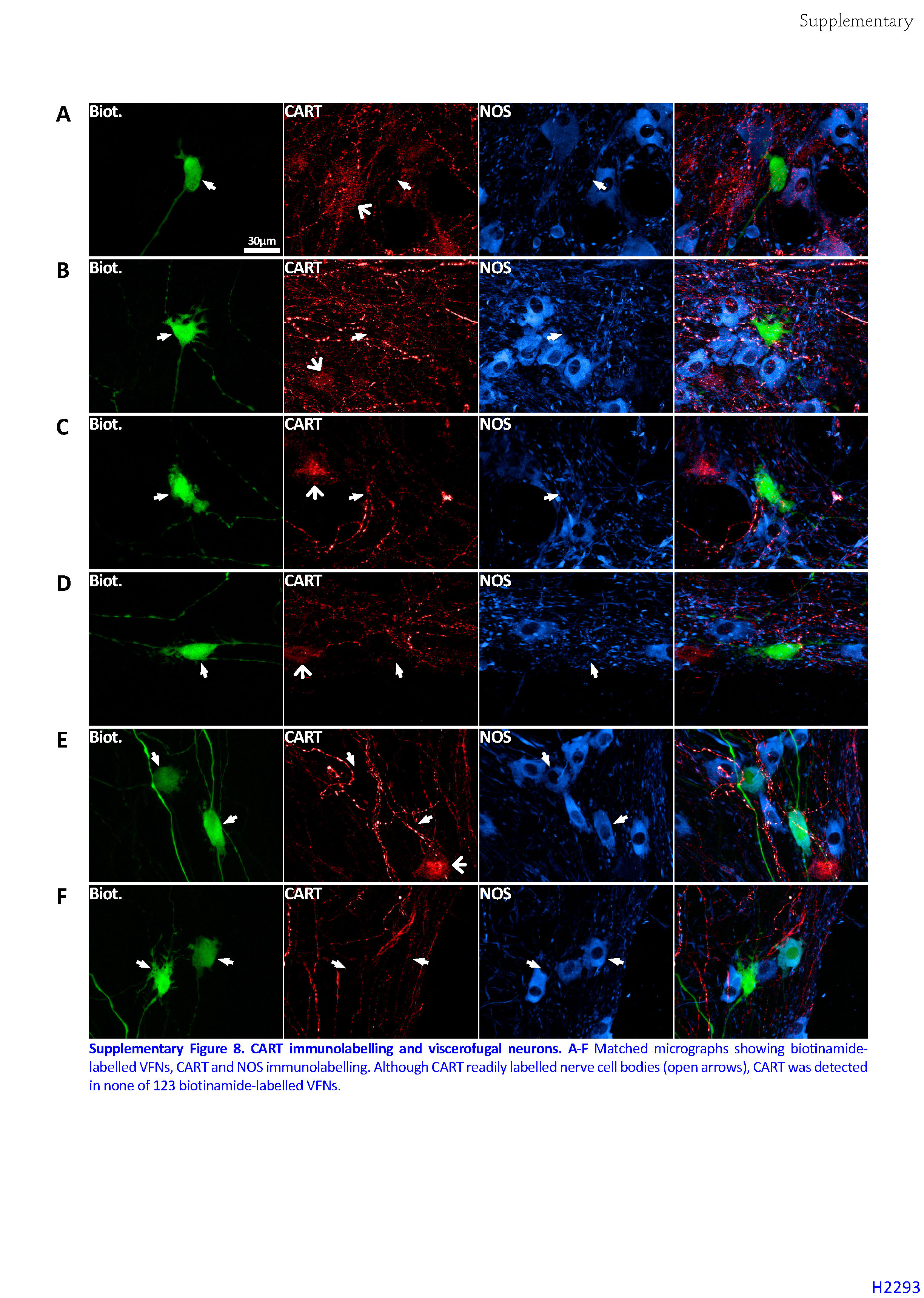

Supplement: Supplementary file 8 [file Image_8.JPEG]

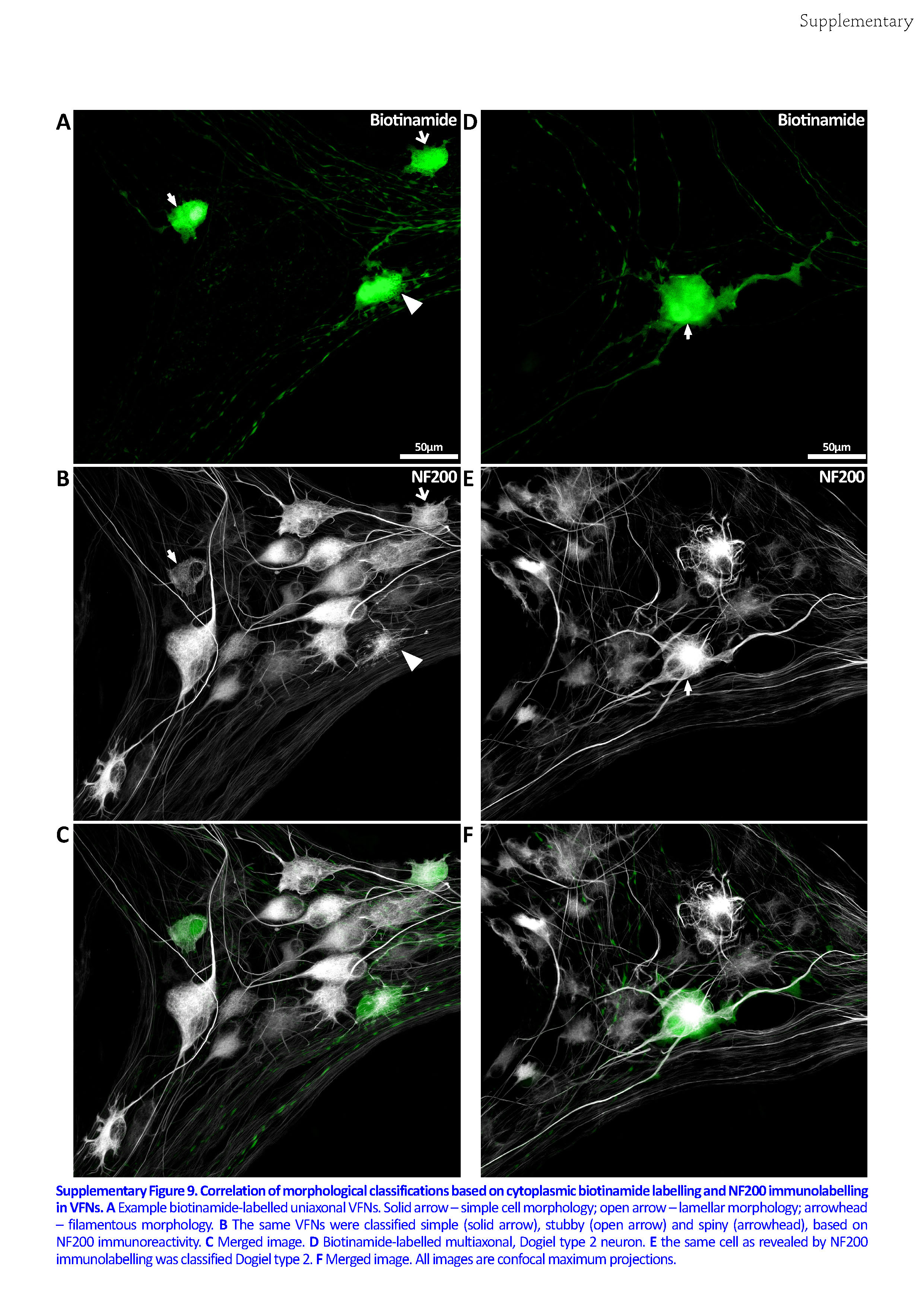

Supplement: Supplementary file 9 [file Image_9.JPEG]
