# Supplementary material for: Characterization of viscerofugal neurons in human colon by retrograde tracing and multi-layer immunohistochemistry
Source: Front Neurosci. 2024 Jan 16;17:1313057. doi: 10.3389/fnins.2023.1313057 (PMC10825022; doi:10.3389/fnins.2023.1313057)
Supplement: Supplementary file 10 [file Table_1.docx]

**Supplementary material**

Supplementary Table 1 – Patients characteristics

| **Prep** | **Region** | **Sex** | **Age** | **Condition** | **VFNs** | **Use** |
| --- | --- | --- | --- | --- | --- | --- |
| 1 | Asc. | F | 33 | Polyps | 38 | Map |
| 2 | Asc. | F | 72 | Cancer | 33 | Map + NCB analysis |
| 3 | Asc. | F | 57 | Cancer | 9 | Map + NCB analysis |
| 4 | Asc. | F | 62 | Cancer | 45 | Map + NCB analysis |
| 5 | Asc. | M | 76 | Cancer | 16 | Map |
| 6 | Asc. | F | 72 | Cancer | 4 | Map |
| 7 | Asc. | M | 54 | Cancer | 56 | Map |
| 8 | Asc. | M | 89 | Cancer | 49 | Map |
| 9 | Desc. | F | 65 | Divert. | 79 | Map + NCB analysis |
| 10 | Desc. | M | 54 | Cancer | 25 | Map + NCB analysis |
| 11 | Desc. | F | 78 | Cancer | 24 | Map + NCB analysis |
| *12* | Desc. | F | 80 | Cancer | 21 | Map |
| *13* | Desc. | F | 80 | Cancer | 5 | Map |
| 14 | Desc. | F | 59 | Cancer | 11 | Map |
| 15 | Desc. | F | 87 | Cancer | 7 | Map |
| *16* | Desc. | F | 64 | Cancer | 47 | Map |
| *17* | Desc. | F | 64 | Cancer | 15 | Map |
| 18 | Desc. | M | 31 | Cancer | 121 | Map |
| 19 | Sig. | F | 87 | Cancer | 30 | Map |
| 20 | Sig. | F | 45 | Cancer | 36 | Map |
| 21 | Sig. | M | 64 | Cancer | 36 | Map |
| 22 | Rect. | F | 53 | Divert. | 6 | Map |
| 23 | Rect. | F | 35 | Cancer | 102 | Map |
| 24 | Rect. | M | 31 | Polyps | 36 | Map |
| 25 | Rect. | F | 31 | Crohn's | 52 | Map |

Note: Preparations 12-13 and 16-17 were derived from the same patient specimen.
