# Supplementary material for: Characterization of viscerofugal neurons in human colon by retrograde tracing and multi-layer immunohistochemistry
Source: Front Neurosci. 2024 Jan 16;17:1313057. doi: 10.3389/fnins.2023.1313057 (PMC10825022; doi:10.3389/fnins.2023.1313057)
Supplement: Supplementary file 11 [file Table_2.docx]

**Supplementary material**

Supplementary Table 2 – Primary and secondary antibodies used for each iteration of immunohistochemical double labelling

|  | Primary |  |  |  |  |  | Secondary |  |  |
| --- | --- | --- | --- | --- | --- | --- | --- | --- | --- |
| Layer | Antigen | Source | Host | Cat No | Dilution | RRID | Tag | Cat No | Dilution |
| 1 | ChAT | Schemann | Rabbit | Yeboah | 1:1000 | AB_2314176 | AF555 | Abcam ab150066 | 1:1000 |
|  | NOS | Emson | Sheep | K205 | 1:5000 | AB_2314957 | AF647 | Jackson 713-605-147 | 1:1000 |
| 2 | Enkephalin | Sera Lab | Mouse | MAS 083c | 1:300 | AB_572247 | Cy3 | Jackson 715-165-151 | 1:200 |
|  | Substance P | ImmunoStar | Rabbit | 20064 | 1:1000 | AB_572266 | AF647 | Jackson 711-605-152 | 1:1000 |
| 3 | CGRP | Peninsula | Rabbit | IHC6006 | 1:2000 | AB_2314156 | AF555 | Abcam ab150066 | 1:1000 |
|  | 5-HT | IncStar | Goat | 20079 | 1:1600 | AB_572262 | AF647 | Jackson 705-605-147 | 1:1000 |
| 4 | VAChT | Synaptic systems | Rabbit | 139 103 | 1:1000 | AB_887864 | AF555 | Abcam ab150066 | 1:1000 |
|  | Somatostatin | Abcam | Rat | ab30788 | 1:500 | AB_778010 | Cy5 | Jackson 712-175-153 | 1:200 |
| 5 | VIP | Walsh | Rabbit | 7913 | 1:2000 | AB_2783533 | AF555 | Abcam ab150066 | 1:1000 |
|  | NPY | Blessing | Sheep | E2210 | 1:2000 | AB_2783534 | AF647 | Jackson 713-605-147 | 1:1000 |
| 6 | Calretinin | SWANT | Goat | CG1 | 1:1000 | AB_10000342 | Cy3 | Jackson 705-165-147 | 1:400 |
|  | Calbindin | SWANT | Rabbit | CB-38 | 1:5000 | AB_10000340 | AF647 | Jackson 711-605-152 | 1:1000 |
| 7 | NF200 | Sigma | Mouse | N0142 | 1:1000 | AB_477257 | Cy3 | Jackson 715-165-151 | 1:200 |
|  | Peripherin | Abcam | Chicken | ab39374 | 1:200 | AB_777207 | AF647 | Jackson 703-605-155 | 1:1000 |
|  | CART | R&D Systems | Mouse | 113612 | 1:400 | AB_2068569 | AF555 | Jackson 715-165-151 | 1:200 |
