# Supplementary material for: Characterization of viscerofugal neurons in human colon by retrograde tracing and multi-layer immunohistochemistry
Source: Front Neurosci. 2024 Jan 16;17:1313057. doi: 10.3389/fnins.2023.1313057 (PMC10825022; doi:10.3389/fnins.2023.1313057)
Supplement: Supplementary file 12 [file Table_3.docx]

**Supplementary material**

Supplementary Table 3: combinations of positive markers in VFNs

| **Combination of Immunoreactive markers** | **Number of VFNs** | **Class** | **Type** |
| --- | --- | --- | --- |
| ChAT+/Calret+/NF200+ | 1 | NEW CLASS | NEW TYPE |
| ChAT+/SP+/ENK+/NF200+ | 5 | AIN1 | AINs |
| ChAT+/ENK+ | 1 | AIN3 | AINs |
| ChAT+/ENK+/NF200+ | 14 | AIN3 | AINs |
| NOS+/ChAT+/NF200+ | 1 | DIN3 | DINs |
| NOS+/ChAT+/Calb+/NF200+ | 6 | DIN3 | DINs |
| NOS+/ChAT+/NF200+/VIP+ | 1 | DIN3 | DINs |
| NOS+/ChAT+/Calb+/NF200+/VIP+ | 1 | DIN3 | DINs |
| NOS+/ChAT+ | 4 | DIN4 | DINs |
| ChAT+/Calb+/NF200+ | 9 | EMN1 | EMNs |
| ChAT+/Calb+/Calret+/NF200+ | 1 | EMN1 | EMNs |
| ChAT+/NF200+ | 60 | EMN2 | EMNs |
| ChAT+/SOM+/NF200+ | 1 | EMN2 | EMNs |
| ChAT+/Calb+ | 7 | EMN3 | EMNs |
| ChAT+ | 68 | EMN4 | EMNs |
| ChAT+/SOM+ | 2 | EMN4 | EMNs |
| NOS+/NF200+/VIP+ | 1 | IMN1 | IMNs |
| NOS+/NF200+ | 2 | IMN2 | IMNs |
| NOS+/VIP+ | 1 | IMN3 | IMNs |
| NOS+ | 12 | IMN4 | IMNs |
| ChAT+/SP+/Calret+/NF200+ | 4 | SN1 | SNs |
| ChAT+/SP+/Calb+/Calret+/NF200+ | 1 | SN1 | SNs |
| ChAT+/SP+/NF200+ | 4 | SN2 | SNs |
| no markers | 5 | Misc | Misc |
| Calb+ | 1 | Misc | Misc |
| NF200+ | 1 | Misc | Misc |
| Calb+/NF200+ | 1 | Misc | Misc |
| **TOTAL** | **215** |  |  |

Supplementary Table 3: A sample of 215 VFNs (n=6) showed 27 different combinations of 12 cell body immunohistochemical markers; all but one of these combinations had been ascribed to a functional class in a recent study{Chen, 2023 in press #61}. Note that for each class immunoreactive markers are shown: all other markers were non-immunoreactive. Table 3 shows all 27 combinations and the classes and types of myenteric neuron that they resemble. Note that 148 of 215 cells had combinations of markers similar to excitatory motor neurons (EMNs)
